# Effectiveness of perindopril/amlodipine fixed-dose combination in the treatment of hypertension: a systematic review

**DOI:** 10.3389/fphar.2023.1156655

**Published:** 2024-02-08

**Authors:** Truong Van Dat, Vo Linh Tu, Le Nguyen Anh Thu, Nguyen Nhat Anh Quang, Van Binh, Nguyen Thi Quynh Nga, Duong Hoang Loc, Tran Thi Hong Nguyen, Dao Ngoc Hien Tam, Hong-Han Huynh, Tran Dinh Trung, Uyen Do, Nguyen Tuan Phat, Dang The Hung, Quang-Hien Nguyen, Nguyen Thi Hai Yen, Le Huu Nhat Minh

**Affiliations:** ^1^ Faculty of Pharmacy, University of Medicine and Pharmacy at Ho Chi Minh City, Ho Chi Minh City, Vietnam; ^2^ Faculty of Traditional Medicine, University of Medicine and Pharmacy at Ho Chi Minh City, Ho Chi Minh City, Vietnam; ^3^ Regulatory Affairs Department, Asia Shine Trading & Service Co. Ltd., Ho Chi Minh City, Vietnam; ^4^ International Master Program for Translational Science, College of Medical Science and Technology, Taipei Medical University, Taipei, Taiwan; ^5^ Faculty of Public Health, Danang University of Medical Technology and Pharmacy, Danang, Vietnam; ^6^ Nelda C. Stark School of Nursing, Texas Woman’s University, Houston, TX, United States; ^7^ Cardiovascular Research Department, Methodist Hospital, Merrillville, IN, United States; ^8^ School of Biomedical Engineering & Imaging Sciences, Faculty of Life Sciences & Medicine, King’s College London, London, United Kingdom; ^9^ International Ph.D. Program in Medicine, College of Medicine, Taipei Medical University, Taipei, Taiwan; ^10^ Research Center for Artificial Intelligence in Medicine, Taipei Medical University, Taipei, Taiwan

**Keywords:** fixed-dose combination, hypertension, blood pressure, efficacy, perindopril, amlodipine, effectiveness

## Abstract

**Background:** Uncontrolled blood pressure is a major risk factor for cardiovascular diseases. Fixed-dose combination (FDC) therapy offers a promising approach to addressing this challenge by providing a convenient single-tablet solution that enhances the effectiveness of blood pressure control. In our systematic review, we assess the effectiveness of perindopril/amlodipine FDC in managing blood pressure.

**Methods:** We conducted a comprehensive search across four primary electronic databases, namely, PubMed, Virtual Health Library (VHL), Global Health Library (GHL), and Google Scholar, as of 8 February 2022. Additionally, we performed a manual search to find relevant articles. The quality of the selected articles was evaluated using the Study Quality Assessment Tools (SQAT) checklist from the National Institute of Health and the ROB2 tool from Cochrane.

**Results:** Our systematic review included 17 eligible articles. The findings show that the use of perindopril/amlodipine FDC significantly lowers blood pressure and enhances the quality of blood pressure control. Compared to the comparison group, the perindopril/amlodipine combination tablet resulted in a higher rate of blood pressure response and normalization. Importantly, perindopril/amlodipine FDC contributes to improved patient adherence with minimal side effects. However, studies conducted to date have not provided assessments of the cost-effectiveness of perindopril/amlodipine FDC.

**Conclusion:** In summary, our analysis confirms the effectiveness of perindopril/amlodipine FDC in lowering blood pressure, with combination therapy outperforming monotherapy and placebo. Although mild adverse reactions were observed in a small subset of participants, cost-effectiveness assessments for this treatment remain lacking in the literature.

## Introduction

Cardiovascular diseases (CVDs) have emerged as the predominant causes of mortality on a global scale, with Vietnam being no exception (NIH, 2021). Among the major risk factors for CVDs, hypertension is the primary cause (Fuchs and Whelton, 2020). According to the WHO, hypertension affects 1.28 billion people globally between the ages of 30 and 79, with a significant prevalence in low- and middle-income countries (Wald et al., 2009).

Fixed-dose combination (FDC) therapy, also known as single-pill combination (SPC) therapy (single pills that contain a combination of two or more active ingredients), appears to be an ideal solution for dealing with the difficulty mentioned previously (González-Gómez et al., 2018; Mancia et al., 2023). Using only one tablet with a combination of formulations can enhance effectiveness in controlling blood pressure (BP) (DiPette et al., 2019; Mancia et al., 2023). Moreover, prescriptions can be augmented to include secondary prevention strategies in hypertensive guidelines, alongside the concurrent use of additional medications to address comorbidities (Murphy et al., 2022). Hence, hypertensive patients may find FDC therapy more patient-friendly, resulting in better adherence to treatment (DiPette et al., 2019). Some authors suggest that the better outcomes of FDC might be due to better medical compliance (Verma et al., 2018). At any stage of treatment, both when starting therapy with a two-drug combination and at any subsequent stage, the use of FDC should be favored (Unger et al., 2020; Mancia et al., 2023). Additionally, even when using generic formulations, FDC is less expensive than free-drug combinations of the same medicines (Tsioufis and Thomopoulos, 2017). Due to the advantages brought by FDC, not only does it decrease the non-compliance rate of medication regimens, but it also assists physicians with prescriptions based on current guidelines (Esba et al., 2021). In 2019, FDC drugs in antihypertensive medications were added to the WHO Model List of Essential Medicines (Organization, 2019). Randomized controlled clinical trials (RCTs) have shown that hypertensive patients using FDC therapy have improved their medication adherence and lowered their rate of CVD risk (BP, heart rate, and cholesterol levels were effectively controlled) (Gnanenthiran et al., 2021). The FDC of perindopril/amlodipine has been proven to provide many health benefits for hypertensive patients who have a higher probability of combining several antihypertensive agents with the aim of achieving the targeted BP (Shirley and McCormack, 2015). No existing systematic reviews were found to assess the effectiveness of perindopril/amlodipine FDC. Therefore, this study aims to summarize the efficacy, safety, and cost-effectiveness of this combination to provide insights for clinical implications and future research.

## Methods

### Protocol and registration

This systematic review adhered to the Preferred Reporting Items for Systematic Reviews and Meta-Analysis (PRISMA) checklist (Moher et al., 2009) (Supplementary Table S1).

### Eligibility criteria

**FIGURE 1:**
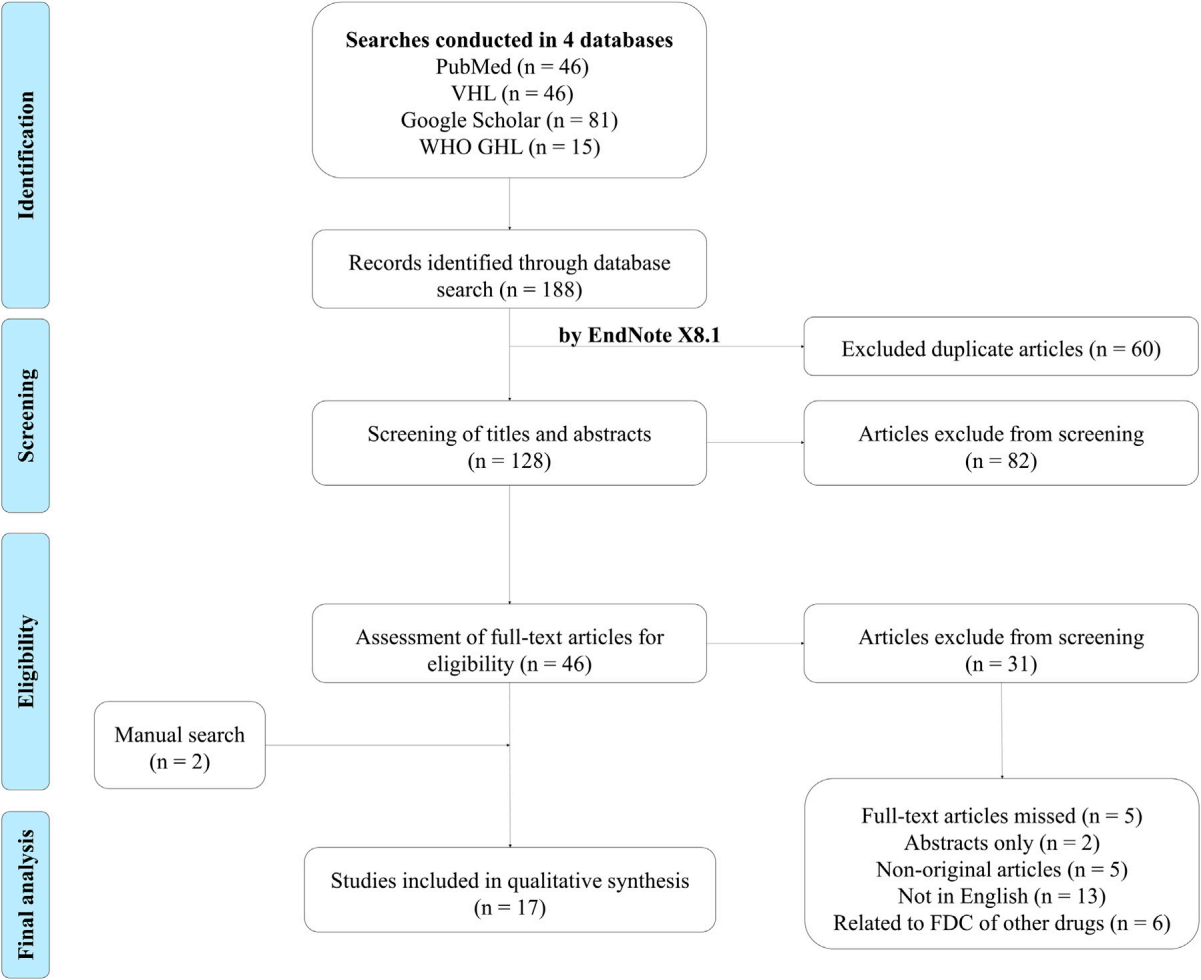
PRISMA flow diagram of study selection.

We selected English studies on the efficacy or cost-effectiveness of perindopril and amlodipine FDC published through 8 February 2022 (no restrictions on the area, study design, or the year of publication). We excluded articles that did not meet our inclusion criteria, specifically those that pertained to FDC drugs containing active ingredients other than perindopril and amlodipine. Additionally, studies conducted on healthy individuals instead of hypertensive patients were removed. Our exclusion list further encompassed book chapters, publications that only provided abstracts, conference reports, reviews, theses, posters, and letters (Figure 1).

### Search strategies

A comprehensive search was conducted across four major databases: PubMed, the Virtual Health Library (VHL), the Global Health Library (GHL), and Google Scholar. We applied filters to narrow our results to research articles focused on humans and written in English. To ensure a thorough review and capture any potentially overlooked studies, we also performed a manual search by examining the references of the articles we included. The specific search terms used in our inquiry can be found in Supplementary Table S2 **.**

### Study selection

Search results from the four aforementioned databases were imported into EndNote X8.1 (Thomson Reuters, CA, United States) for automatic deletion of duplicates. We selected articles in two phases: 1. title and abstract screening of all searched articles; 2. full-text screening and the selection of articles. These two phases of selection were conducted by at least two independent reviewers in accordance with our inclusion and exclusion criteria. A third reviewer carefully addressed any points of disagreement before deciding. The articles selected had no limitation on geography, culture, or ethnicity.

### Data collection process and data items

Data extraction was performed by two independent reviewers, and disagreements between the reviewers were resolved by discussion with a third reviewer to reach a consensus. The extracted data included basic information such as year of publication, study design, number of patients, follow-up period, and baseline patient characteristics. Data were extracted with no statistical modifications and grouped according to each research objective:

- For efficacy data: The efficacy of perindopril/amlodipine FDC in the treatment of hypertension includes changes in blood pressure, rate of hypertension control, reduction in stroke rate, mortality, safety, and adherence to medication.
- For cost-effectiveness data, cost-effectiveness evaluation parameters (e.g., ACER and ICER) will be reported along with research objectives and methods/techniques used.

### Quality assessment

Two independent reviewers assessed the quality of the included studies to determine their risk of bias, and a third reviewer was consulted in case of any disagreements. We employed the quality assessment tool for observational cohort and cross-sectional studies to evaluate the quality of the articles included (the NIH tool for quality assessment can be accessed at the following link: https://www.nhlbi.nih.gov/health-topics/study-quality-assessment-tools).

## Results

### Systematic search, study selection, and study characteristics

As a result, 188 articles were identified in four databases. After excluding all duplicates using EndNote X8.1, there were 128 articles that had potentially relevant articles. The selection of titles and abstracts resulted in 46 articles, which were subsequently analyzed into full texts by the reviewers. Seventeen papers were qualified for the systematic review after the studies that did not match the inclusion criteria were eliminated.

There were 11 prospective observational studies, two retrospective studies, and four double-blind randomized controlled trials. The included studies are summarized in Supplementary Table S3.

### Main results

#### BP and clinical outcomes in non-comparative studies

Eleven prospective observational studies and one analytical study on retrospective data were extracted. Most studies examined the therapeutic efficacy of perindopril/amlodipine FDC at doses of 5/5, 5/10, 10/5, and 10/10 mg in untreated or uncontrolled hypertensive patients with previous treatment. Table 1 shows the effectiveness in reducing BP and increasing the BP control rates in the total hypertensive population of the studies (Nagy, 2013; Manolis et al., 2015; Abdelhady et al., 2016; Forster and Dézsi, 2016; Vlachopoulos et al., 2016; Liakos et al., 2017; Fleig et al., 2018).

**TABLE 1:** BP and clinical outcomes in non-comparative studies of perindopril/amlodipine FDC at doses of 5/5, 5/10, 10/5, and 10/10 mg.

Study	S: single/M: multi-center^##^	Observation duration	Pre-BP (SBP/DBP mmHg), mean ± SD	Post-BP (SBP/DBP mmHg), mean ± SD	Controlled BP^**#**^ (% no. patients)	*p*-value
H03 (Liakos et al*.*,^2017^)	S (Greece)	4 months	156.5 ± 15.0/89.9 ± 9.6	130.8 ± 8.4/78.2 ± 6.4	81.5	*p* < 0.001
H17 (Vlachopoulos et al., 2016)	M	4 months	158.4 ± 13.6/89.9 ± 8.7	130.0 ± 7.9/77.7 ± 6.3	88.5	*p* < 0.001
H07 (Manolis et al., 2015)	S (Greece)	6 months	157.0 ± 15.4/91.5 ± 10.1	129.0 ± 7.9/78.8 ± 6.7	84.8	*p* < 0.001
H37 (Forster and Dézsi, 2016)	M	6 months	157.5 ± 12.9/92.9 ± 8.6	130.3 ± 8.3/79.8 ± 6.1	80.6	*p* < 0.0001
H46 (Nagy, 2013)	M	3 months	159.8 ± 16.0/94.3 ± 10.3	131.1 ± 10.2/80.0 ± 7.1	82	*p* < 0.001
H15 (Fleig et al., 2018)	S (Germany)	3 months	163.7 ± 14.8/95.4 ± 9.4	133.6 ± 11.6/80.3 ± 7.7	69.1	*p* < 0.0001
H41 (Abdelhady et al., 2016)	S (Saudi Arabia)	4 weeks	161.7 ± 13.9/99.4 ± 8.4	141.8 ± 12.2/88.7 ± 7.9	93.3	*p* < 0.0001
		8 weeks		132.4 ± 10.3/83.2 ± 6.7		*p* < 0.0001
		12 weeks		127.6 ± 8.6/80.1 ± 5.4		*p* < 0.0001

Here is a revised legend for the table:

“Data are displayed as mean ± SD. BP represents blood pressure, with SBP representing systolic blood pressure and DBP representing diastolic blood pressure. Blood pressure readings are presented in the format of SBP/DBP. The symbol “#” indicates the count of patients whose blood pressure was effectively managed post-treatment, referred to as “controlled BP.” “##” denotes the country of the single center in cases where applicable.”

Other studies have also shown evidence of perindopril/amlodipine FDC efficacy. In an analysis of two studies in hypertensive patients switching from ACE inhibitors and/or CCBs to the applicable dose of one perindopril/amlodipine FDC, the prospective cohort data analysis also demonstrated the efficacy of perindopril/amlodipine FDC with an increase in the BP target rate from 16.0% to 50.6% (Hatalova et al., 2016); additionally, Nagy (2013) showed that after 3 months, the study population’s mean 24-h ambulatory BP decreased from 146.1/84.3 to 127.6/75.9 mmHg (*p* < 0.001) (Nagy, 2013).

Treatment with perindopril/amlodipine FDC (4 mg/5 mg) in 73 stage 2 hypertensive patients reduced BP by 35.2 [31.7–38.7]/20.6 [19.2–22.1] mmHg [mean (range)], and BP control reached 57.3%. Among stage 3 hypertensive patients, treatment with perindopril/amlodipine FDC (4 mg/5 mg) (*n* = 18) resulted in a substantial reduction in blood pressure, with a decrease of 46.1 [40.1–52.3]/23.8 [19.9–27.7] mmHg, achieving a blood pressure control rate of 27.8% (Bansal et al., 2014). Furthermore, Fleig et al. (2018) also showed that patients with stage 2 or 3 hypertension decreased from 64.4% to 3.9%; of those with isolated diastolic hypertension, 67.6% returned to normal index.

Karpov et al. (2015) showed that perindopril/amlodipine FDC (5/5, 10/5, 10/10 mg) acted rapidly (2 weeks) and significantly reduced BP after 3 months in the clinic (−33.7/17.1 mmHg, *p* < 0.001) in patients at high or extremely high cardiovascular risk. Ambulatory and self-monitored blood pressure rates significantly decreased throughout the study period (*p* < 0.0001).

When comparing hypertensive patients with and without diabetes, the rate of BP control achieved was 87% of patients, with no significant difference between groups (86% and 88%, *p* = 0.499), and BP decreased by 31/18 mmHg (*p* < 0.001) after 3 months of using perindopril/amlodipine FDC (Ahmed et al., 2016). The mean proportion of patients with controlled BP was 93.3% at week 12, of which the proportion of patients without diabetes was 96.4% and of patients with diabetes was 89.0% (Abdelhady et al., 2016).

The study investigated the long-term efficacy and mortality of perindopril/amlodipine FDC compared with a two-pill combination in the treatment of hypertension. After 48 months, the mortality rate was 8% in the FDC group and 18% in the combination group. The hazard ratio for the risk of death for the combination drug compared with FDC was 2.81 (95%, CI 2.42–3.26) (unadjusted) and 1.83 (95%, CI 1.55–2.16) (adjusted for potential confounding variables) (Simons et al., 2017).

In addition, Forster and Dézsi (2016) indicated that treatment with FDC perindopril/amlodipine had a cardioprotective effect. The Canadian Cardiovascular Society (CCS) index has moved in a positive direction (*p* < 0.0001). In patients with an ECG exercise stress test (n = 197), it was found that the patient’s mobility increased significantly after 6 months, the maximum efficiency increased from 88.9 ± 37.9 W to 110.5 ± 38.4 W (24.4%; *p* < 0.001), and the metabolic equivalent of task (MET) increased from 7.86 ± 2.95 to 8.78 ± 2.92 (11.7%; *p* < 0.001) (Forster and Dézsi, 2016).

In summary, all 12 studies showed that treatment with perindopril/amlodipine combination tablets significantly reduced BP and increased the rate of BP control. The aforementioned studies have encouraged the use of perindopril/amlodipine FDC.

#### BP and clinical outcomes in comparative studies

There were four randomized, double-blind, controlled clinical trials, of which one included two phase III clinical trials conducted in China, one study was conducted in 18 countries, one study was conducted in six countries in Europe, and one study was conducted in Cameroon. Studies compared the efficacy of perindopril/amlodipine FDC with perindopril or amlodipine monotherapy, perindopril/indapamide FDC, valsartan/amlodipine FDC at various doses, and placebo. Table 2 shows the characteristics of the randomized, double-blind, controlled clinical studies.

**TABLE 2:** BP and clinical outcomes in comparative studies.

Study	Country of study	Baseline	Case group			Baseline	Control group			Follow-up period
		BP baseline	N_1_	Medication	∆BP^#^ (mmHg)	BP baseline	N_2_	Medication	∆BP^#^ (mmHg)	
H21 (Hu et al., 2016)	China	149.6 ± 6.9/96.9 ± 4.6	247	Per/Amlo (5/5; 5/10 mg)	−11.1 ± 11.9/-	150.5 ± 7.4/96.4 ± 4.4	245	Amlodipine 5 mg	−8.5 ± 11.1/-	8 weeks
		150.7 ± 6.8/97.6 ± 5.0	178	Per/Amlo (5/5 mg)	−15.8 ± 12.1/-	150.0 ± 6.8/97.7 ± 4.7	175	Perindopril 4 mg	−7.8 ± 13.7/-	8 weeks
H38 (Sobngwi et al., 2019)	Cameroon	143(140–150)/91(85–93)	15	Per/Amlo (5/5 mg)	−16/19	147 (141–151)/89 (84–96)	15	Perindopril/indapamide (5/1.25 mg)	−23/11	42 days
H36 (Laurent et al., 2015)	France, Russia, Ukraine, Latvia, Lithuania, Hungary	100.7 ± 4.0/161.8 ± 7.5	248	Per/Amlo (3,5/2.5 mg)	−22.0 ± 14.0/13.6 ± 9.2	160.9 ± 7.3/100.5 ± 3.9/	250	Placebo	−14.2 ± 16.1/9.3 ± 9.2	2–3 weeks
						100.7 ± 4.0/161.5 ± 7.8	273	Perindopril 3.5 mg	−16.3 ± 17.0/9.7 ± 9.9	8 weeks after the placebo
						161.0 ± 7.6/10.6 ± 4.0	274	Amlodipine 2.5 mg	−16.0 ± 15.3/10.3 ± 9.7	
						160.7 ± 7.3/100.1 ± 4.1	272	Perindopril 5 mg	−18.2 ± 14.8/10.5 ± 9.7	
						162.3 ± 7.5/100.6 ± 4.0	264	Amlodipine 5 mg	−21.8 ± 15.4/12.6 ± 8.9	
MS3 (Mancia et al., 2015)	Eighteen countries	163.6 ± 7.9/100.2 ± 3.7	881	Per/Amlo	−25.9 ± 13.3/16.9 ± 8.7	163.4 ± 8.0/100.2 ± 3.8	876	Valsartan/amlodipine	−23.6 ± 14.2/15.5 ± 9.2	3 months

Data are displayed as mean ± SD.

The abbreviations used are as follows: BP, blood pressure; SBP, systolic blood pressure; DBP, diastolic blood pressure. Blood pressure measurements are presented in the format of SBP/DBP. N denotes the number of patients, while ∆BP represents the change in blood pressure from the baseline. Per/Amlo stands for perindopril and amlodipine.

The study consisted of two trials comparing the efficacy of perindopril/amlodipine FDC with amlodipine or perindopril monotherapy, named 016 and 017, respectively. After 8 weeks, the mean SBP in the perindopril/amlodipine FDC (5/5 mg) showed a mean reduction of 11.1 ± 11.9 mmHg compared to a mean reduction of 8.5 ± 11.1 mmHg SBP in the amlodipine 5-mg group (*p* = 0.0095). Mean SBP decreased to 15.8 ± 12.1 mmHg in the perindopril/amlodipine FDC group compared with 7.8 ± 13.7 mmHg in the perindopril 4-mg group (*p* < 0.0001) (Hu et al., 2016).

Thirty patients were randomly divided into two groups, with one receiving the perindopril/amlodipine FDC (5/5 mg) and the other receiving perindopril/indapamide (5/1.25 mg). After a 6-week period, the perindopril/amlodipine group experienced a decline in SBP from 144 mmHg to 128 mmHg (*p* = 0.03). In comparison, the perindopril/indapamide group saw a drop from 147 mmHg to 124 mmHg (*p* = 0.008). Furthermore, the perindopril/amlodipine group’s DBP (presumed from the data) decreased from 91 mmHg to 72 mmHg (*p* = 0.001), whereas the perindopril/indapamide group’s DBP reduced from 89 mmHg to 78 mmHg (*p* = 0.008). Over a 24-h period, the perindopril/amlodipine group’s SBP went from 144 mmHg to 128 mmHg (*p* = 0.003), while it decreased from 145 mmHg to 126 mmHg in the perindopril/indapamide group (*p* = 0.003). Additionally, the 24-h SBP in the perindopril/amlodipine group decreased from 85 mmHg to 78 mmHg (*p* = 0.013), as compared to a reduction from 89 mmHg to 79 mmHg in the perindopril/indapamide group (*p* = 0.006) (Sobngwi et al., 2019).

An international, randomized, double-blind, placebo-controlled study of patients with mild-to-moderate hypertension was conducted at 188 centers in six European countries. The clinical outcome of perindopril/amlodipine FDC was significantly superior to placebo (between-group difference: 7.22/4.12 mmHg; *p* < 0.001). Response rates and BP normalization were higher in the FDC group (*p* < 0.001 for both), and a significant difference from the placebo was evident at 2 weeks. In addition, FDC was superior to either component administered alone (*p* < 0.001) (Laurent et al., 2015).

Randomized controlled clinical trials were performed in 194 centers in 18 countries, comparing the efficacy and safety of dose escalation with perindopril/amlodipine FDC and the dose escalation regimen with valsartan/amlodipine FDC. The study’s results showed that the treatment of mild and moderate hypertension started with perindopril/amlodipine FDC (3.5/2.5 mg), and then the dose was increased to 7/5 and 14/10 mg, giving the most effective results and achieving better BP regulation than other valsartan regimens. In comparison to valsartan/amlodipine, perindopril/amlodipine achieved a rate of controlled hypertension after 1 month of treatment of 33% *versus* 27% (estimated difference: 6.1%; *p* = 0.005). After 3 months, compared to treatment with valsartan/amlodipine FDC, BP decreased more than baseline with perindopril/amlodipine FDC (between-group difference: −2.0/-1.5 mmHg; *p* < 0.001). All other visits produced comparable results (*p* ≤ 0.001) (Mancia et al., 2015). All four studies showed that using the perindopril/amlodipine combination tablet resulted in a higher rate of response and normalization of BP compared with the comparison group.

#### Safety

There were 14 studies that reported the safety of perindopril/amlodipine FDC (Supplementary Table S2). Hu et al. reported emergent adverse events (EAEs) of perindopril/amlodipine FDC, including stroke (one patient), synovitis (one patient), pregnancy (one patient), cataracts (one patient), and diaphragmatic complications (one patient). While serious EAEs in the comparator drug group included lumbar intervertebral disc protrusion (one patient), cerebral infarction (one patient), and lacunar infarction (one patient) (Hu et al., 2016). In the study by Fleig et al., only eight patients (0.4%) experienced severe EAEs in 140 ADR of 88 patients, of which four patients (0.2%) did not recover and one patient died with a hypertensive crisis (Fleig et al., 2018).

EAEs that were not serious, including dry cough, ankle and lower extremity edema, facial flushing, hypotension, constipation, and allergy, may have resulted in some patients withdrawing from the study (Manolis et al., 2015; Hu et al., 2016; Vlachopoulos et al., 2016). The majority of EAEs were more frequent, and some were more severe than the comparison group.

Bansal et al. (2014) reported a dry cough that interfered with the patient’s sleep. The incidence of this event was 1/140 (1.8%) among people using the FDC of perindopril and indapamide and 4/93 (4.3%) among people using FDC of perindopril and amlodipine. The rate of cough leading to discontinuation of treatment was 1/10 (0.1%). In other studies, the incidence of patients experiencing dry cough while using perindopril/amlodipine FDC was reported as 1/13 in the study by Liakos et al. (2017), 4/25 in Vlachopoulos et al. (2016), 1/7 in Nagy (2013), 5% in Abdelhady et al. (2016), and 0.2% in Forster and Dézsi (2016).

The reported incidence of ankle edema ranges from 0.6% to 16% (Bansal et al., 2014; Abdelhady et al., 2016; Forster and Dézsi, 2016). Hatalova et al. (2016) showed that the risk associated with ankle edema, compared with previous treatment with ACEIs and/or CCBs, was −37.5% in the first month (*p* < 0.001) and −57.2% in the third month (*p* < 0.001).

The rate of adverse drug reactions in the perindopril/amlodipine group was higher than that in the placebo group (18.9% vs. 15.9%), and it was comparable to the group using perindopril (3.5 mg) and amlodipine (2.5 mg) (18.9% vs. 18.7% and 18.6%) and was highest in the group of amlodipine (5 mg) (21.6%). The group of patients using FDC did not experience serious adverse drug reactions compared with the group using perindopril (5 mg) and amlodipine (5 mg) (Laurent et al., 2015).

In addition, a small percentage of patients reported other side effects such as hypotension (Forster and Dézsi, 2016; Vlachopoulos et al., 2016), extra systolic arrhythmia (Liakos et al., 2017), headache, and dizziness (Abdelhady et al., 2016).

#### Drug adherence

There were seven studies that reported an increase in adherence in patients with perindopril/amlodipine FDC. Fleig et al. (2018) showed that using FDC improves life quality and medication adherence. Most patients were compliant with treatment “every day” or “quite often” (Manolis et al., 2015; Vlachopoulos et al., 2016). The rate of patients who adhered to medication also increased compared to previous treatment (Karpov et al., 2015; Degli Esposti et al., 2018). In addition, after 12 months, the rate of patients stopping treatment on FDC was 34%, less than the rate of patients taking two separate tablets, at 57% (Simons et al., 2017). Supplementary Table S4 provides a summary of the studies.

#### Cost-effectiveness

Studies to date have not provided the results of perindopril/amlodipine FDC cost-effectiveness assessment.

### Quality assessment

Cohort and cross-sectional studies were evaluated using the Study Quality Assessment Tools (SQAT) (NIH, 2021) by the National Institute of Health, and RCTs were evaluated using Cochrane’s ROB2 (Higgins et al., 2011). Regarding the overall risk of bias judgment, all four RCTs were of some concern due to adherence to the intervention (Supplementary Table S5). Of the 13 observational studies, 12 had a good rating and the other one had a fair rating (Supplementary Table S6).

## Discussion

This review included a total of 17 studies that investigated the efficacy of perindopril/amlodipine FDC hypertension treatment. In general, this combination showed significant effects on lowering BP. Both ACC/AHA and ESC/ESH recommended treatment with ACE inhibitors, or ARBs, or diuretics, or CCBs. Although monotherapy was sometimes effective, most patients needed additional drugs afterward (Wald et al., 2009). A two-drug combination (ACEi or ARB and diuretic or CCB) was preferable, indicated by both ACC/AHA and ESC/ESH to reach a BP target <130/80 mmHg (Whelton et al., 2022). Perindopril is an ACE inhibitor, whereas amlodipine is a CCB. In 2015, a fixed dosage of these drugs was approved by the FDA with three strengths (3.5/2.5, 7/5, and 14/10 mg) (Shirley and McCormack, 2015). In this review, we recognized that this combination with a variety of dosages was remarkably effective in lowering SBP by over 20 mmHg (Karpov et al., 2015; Fleig et al., 2018). Six of seven studies demonstrated a BP control rate of over 70%, indicating the efficacy of perindopril/amlodipine FDC [H03, H17, H07, H37, H46, and H41]. Compared to the valsartan/amlodipine (ARB/CCB) FDC strategy, the perindopril/amlodipine (ACEi/CCB) FDC strategy proved significantly better BP-lowering outcomes and fewer up-titration steps (Higgins et al., 2011; Degli Esposti et al., 2018). Nonetheless, it is imperative to highlight that the perindopril/amlodipine FDC strategy was initiated with the combination from the outset, whereas the valsartan/amlodipine FDC strategy commenced with valsartan monotherapy. Therefore, it is crucial to interpret the outcomes of this study within the context of the entire treatment strategy rather than as a direct comparison of efficacy between ARB/CCB and ACEi/CCB. Moreover, it is noteworthy that the effectiveness of blood pressure (BP) reduction appeared to exhibit a remarkable similarity between perindopril/amlodipine (ACEi/CCB) and perindopril/indapamide (ACEi/thiazide diuretic), suggesting the potential interchangeability of these combination therapies (Sobngwi et al., 2019). This finding aligns with the recommendations of the ACC/AHA and ESC/ESH guidelines, which endorse the use of combination therapy involving these classes of medication. Furthermore, the treatment with perindopril/amlodipine FDC demonstrated a reduction in CVD risk, as indicated by measures such as the CCS ratio, ECG outcomes, and MET (Forster and Dézsi, 2016).

One of the advantages of a single-pill FDC is the reduction in the number of drugs patients need to take, consequently leading to improved adherence. Four studies included in this review demonstrated favorable adherence to the treatment, which may contribute to enhanced blood pressure control (Karpov et al., 2015; Manolis et al., 2015; Vlachopoulos et al., 2016; Degli Esposti et al., 2018). Furthermore, patients who were prescribed a single-pill regimen appeared to encounter fewer adverse events when compared to those on individual component regimens (Belsey, 2012). The rates of reported side effects in the included studies were relatively low, spanning a range of 1.8%–18.9% (Bansal et al., 2014; Laurent et al., 2015). Cough were reported as the most frequent adverse events associated with the use of perindopril/amlodipine FDC, ranged from 4.6% to 9.0% (Hu et al., 2016). Most patients tolerate cough when continuing treatment, with only a few requiring discontinuations.

On the other hand, no cost-effective study meeting the inclusion criteria has been identified. Several non-English reports did provide cost-effectiveness data concerning perindopril/amlodipine FDC; however, these data exhibited inconsistencies. For instance, a Chinese report indicated that the daily cost per patient and the annual cost per patient of perindopril arginine/amlodipine (in various tablet strengths: 3.5 mg/2.5 mg, 7 mg/5 mg, and 14 mg/10 mg) ranged from 0.95 to 1.15 dollars and 347 to 420 dollars, respectively. Notably, the FDC of perindopril arginine/amlodipine, with an annual per-patient cost of 347–420 dollars, was found to be more expensive than the individual components of perindopril erbumine (with an annual per-patient cost ranging from 238 to 413 dollars) and amlodipine (with an annual per-patient cost ranging from 50 to 131 dollars). Additionally, the total cost of perindopril arginine/amlodipine exceeded that of ACE inhibitors, ARBs, and calcium channel blockers when used as monotherapy. However, the cost of perindopril arginine/amlodipine was lower than that of other combinations such as ACE inhibitors/CCBs (verapamil/trandolapril with an annual cost ranging from 629 to 698 dollars) but higher in price than ARB/CCB combinations (telmisartan/amlodipine with an annual cost of 256 dollars). Its cost was also lower than combinations of CCBs and ACE inhibitors (annual cost per patient ranging from 160 to 887 dollars), ARBs and CCBs (annual cost per patient ranging from 208 to 953 dollars), and CCBs/β-blockers (annual cost per patient ranging from 131 to 872 dollars); however, the cost variations were highly dependent on the specific combination used. Another Chinese report indicated that the treatment cost of perindopril/amlodipine was 6.21 yuan at a hospital, which was lower than the cost of single-pill perindopril and amlodipine (10.3 yuan). The cost-effectiveness ratios of the FDC groups consistently demonstrated lower values than other groups (Lu, 2020). A study based on the potential program in Russia explored the effectiveness of combining perindopril and amlodipine in the treatment of hypertension. The inclusion of Prestance (perindopril arginine/amlodipine) alongside conventional hypertension treatment resulted in a significant reduction in total costs, ranging from 5 to 5.8 times lower. Over a span of 5 years, effective cost management of medication usage could potentially decrease the overall cost of hypertension treatment and associated ischemic stroke by 1.39 to 1.46 times (Dyakov and Glezer, 2016).

Several studies have explored the cost-effectiveness of FDC compared to free combination therapy (FrCT). Generally, while FDC may have a higher cost for the drug itself, it contributes to better cost-effectiveness at the system level. For instance, Ferrario et al. (2013) found that mean monthly unadjusted all-cause healthcare costs for individuals prescribed single pill amlodipine/benazepril FDC (unadjusted cost was 780 dollars) or single pill amlodipine/olmesartan FDC (unadjusted cost was 740 dollars) were lower than that for the group receiving amlodipine and an ARB in the FrCT (unadjusted cost was 1,394 dollars). This suggests that single pill FDC regimens may offer cost savings when compared to FrCT counterparts. In the study by Bramlage et al. (2018), it was demonstrated that the annual cost per patient of FDC therapy was higher than that of FrCT. Specifically, for ramipril/amlodipine FDC, the annual cost per patient was 230.2 euros, whereas for FrCT, it was 134.16 euros (*p* < 0.001). Similarly, for candesartan/amlodipine FDC, the annual cost per patient was 339.61 euros, whereas for FrCT, it was 235.01 euros (*p* < 0.001). After adjusting for factors such as age, gender, and comorbidities, the annual cost difference was +98.9 euros for ramipril/amlodipine FDC and +107.1 euros for candesartan/amlodipine FDC, indicating that FDC therapy was associated with higher costs than FrCT. However, this research also showed that the persistence and adherence with FDC were higher than FrCT, which may decrease the cost for outpatient care, emergency visits, and hospital admissions, thereby facilitating better financial outcomes. A study conducted by Wang et al. in Texas, using a Markov model over a span of 5 years, reported that for the prevention of cardiovascular events, single-pill triple combination therapy was more cost-effective than FrCT in the strict adherence measurement definition model. The incremental cost-effectiveness ratio (ICER) or the cost per quality-adjusted life year (QALY) gained for the single-pill FDC was $33,862.46, compared to the FrCT group. In the relaxed adherence measurement definition model, the ICER of single-pill FDC compared to FrCT was $84,932.26. Using a willingness-to-pay threshold of $50,000, single-pill FDC was shown to be more cost-effective than FrCT if strict adherence measurement was used. Notably, in both strict and relaxed definition models, the QALYs of single-pill FDC compared to FrCT increased by 2.26 and 0.67 years, respectively (Wang et al., 2021).

One significant limitation of our study is the availability of control groups in only six out of the 17 publications included in our analysis. This limitation arises from the inherent heterogeneity among these control groups, rendering them unsuitable for meta-analysis. As a result, our ability to draw comprehensive conclusions and conduct statistical comparisons across the entire dataset is restricted. This limitation will be duly acknowledged in our paper to ensure transparency and to highlight the potential impact on the scope of our findings.

## Conclusion

In conclusion, the cumulative findings derived from the included research papers provide compelling evidence supporting the efficacy of perindopril/amlodipine FDC in reducing blood pressure levels. Moreover, our analysis underscores the superior effectiveness of the combination of active ingredients found in perindopril/amlodipine SPCs when compared to both monotherapy and placebo. Although there were instances of a slightly elevated occurrence of adverse drug reactions associated with the combination therapy, it is noteworthy that our research reveals that these side effects were mild and affected only a limited percentage of participants. It is important to acknowledge that our study did not uncover any assessments of the cost-effectiveness of perindopril/amlodipine FDC within the existing literature.

## Article contribution

Uncontrolled blood pressure represents a significant risk factor for cardiovascular disease. Utilizing fixed-dose combination (FDC) therapy shows potential to enhance the effectiveness of blood pressure (BP) control. In our study, we systematically reviewed evidence from four primary electronic databases, namely, PubMed, the Virtual Health Library (VHL), the Global Health Library (GHL), and Google Scholar, as of 8 February 2022, to assess the effectiveness of the perindopril/amlodipine FDC.

Our analysis, based on 17 included articles, demonstrates that the use of perindopril/amlodipine FDC significantly reduces blood pressure and enhances the quality of BP control. The perindopril/amlodipine combination tablet is associated with a higher rate of BP response and normalization when compared to the comparison group. Importantly, perindopril/amlodipine FDC improves patient adherence while exhibiting minimal side effects. These findings highlight the effectiveness of perindopril/amlodipine fixed-dose combination therapy in real-life clinical settings, not only in reducing blood pressure but also in enhancing patient adherence.
