# Corrigendum: Effectiveness of perindopril/amlodipine fixed-dose combination in the treatment of hypertension: a systematic review

**DOI:** 10.3389/fphar.2024.1426608

**Published:** 2024-06-04

**Authors:** Truong Van Dat, Vo Linh Tu, Le Nguyen Anh Thu, Nguyen Nhat Anh Quang, Van Binh, Nguyen Thi Quynh Nga, Duong Hoang Loc, Tran Thi Hong Nguyen, Dao Ngoc Hien Tam, Hong-Han Huynh, Tran Dinh Trung, Uyen Do, Nguyen Tuan Phat, Dang The Hung, Quang-Hien Nguyen, Nguyen Thi Hai Yen, Le Huu Nhat Minh

**Affiliations:** ^1^ Faculty of Pharmacy, University of Medicine and Pharmacy at Ho Chi Minh City, Ho Chi Minh City, Vietnam; ^2^ Faculty of Traditional Medicine, University of Medicine and Pharmacy at Ho Chi Minh City, Ho Chi Minh City, Vietnam; ^3^ Regulatory Affairs Department, Asia Shine Trading & Service Co. Ltd., Ho Chi Minh City, Vietnam; ^4^ International Master Program for Translational Science, College of Medical Science and Technology, Taipei Medical University, Taipei, Taiwan; ^5^ Faculty of Public Health, Danang University of Medical Technology and Pharmacy, Danang, Vietnam; ^6^ Nelda C. Stark School of Nursing, Texas Woman’s University, Houston, TX, United States; ^7^ Cardiovascular Research Department, Methodist Hospital, Merrillville, IN, United States; ^8^ School of Biomedical Engineering & Imaging Sciences, Faculty of Life Sciences & Medicine, King’s College London, London, United Kingdom; ^9^ International Ph.D. Program in Medicine, College of Medicine, Taipei Medical University, Taipei, Taiwan; ^10^ Research Center for Artificial Intelligence in Medicine, Taipei Medical University, Taipei, Taiwan

**Keywords:** fixed-dose combination, hypertension, blood pressure, efficacy, perindopril, amlodipine, effectiveness

In the published article, there was an error in **Affiliation 1**. Instead of “Department of Pharmacy, University of Medicine and Pharmacy at Ho Chi Minh City, Ho Chi Minh city, Vietnam,” it should be “Faculty of Pharmacy, University of Medicine and Pharmacy at Ho Chi Minh City, Ho Chi Minh City, Vietnam.”

In the published article, there was an error regarding the **Affiliation** for Tran Dinh Trung. Instead of “Department of Pharmacy, University of Medicine and Pharmacy at Ho Chi Minh City, Ho Chi Minh city, Vietnam”, it should be “Faculty of Public Health, Danang University of Medical Technology and Pharmacy, Danang, Vietnam.”

In the published article, there was an error.

A correction has been made to **Results**, *Main results*, Safety, Paragraph Number 1. This paragraph previously stated:

“There were 14 studies that reported the safety of perindopril/amlodipine FDC (**Supplementary Table S2**). Of these, several serious but rare emergent adverse events (EAEs) of perindopril/amlodipine FDC have been reported, including stroke, brain infarction, cataracts, and diaphragmatic eventration (**Hu et al., 2016**). In the study by Fleig et al., eight patients (0.4%) experienced severe EAEs in 140 ADR of 88 patients, of which four patients (0.2%) did not recover and one patient died with a hypertensive crisis (**Fleig et al., 2018**).”

The corrected sentence appears below:

“There were 14 studies that reported the safety of perindopril/amlodipine FDC (**Supplementary Table S2**). Hu et al. reported emergent adverse events (EAEs) of perindopril/amlodipine FDC, including stroke (one patient), synovitis (one patient), pregnancy (one patient), cataracts (one patient), and diaphragmatic complications (one patient). While serious EAEs in the comparator drug group included lumbar intervertebral disc protrusion (one patient), cerebral infarction (one patient), and lacunar infarction (one patient) (**Hu et al., 2016**). In the study by Fleig et al., only eight patients (0.4%) experienced severe EAEs in 140 ADR of 88 patients, of which four patients (0.2%) did not recover and one patient died with a hypertensive crisis (**Fleig et al., 2018**).”

The authors apologize for these errors and state that this does not change the scientific conclusions of the article in any way. The original article has been updated.

