# Corrigendum: Effectiveness of perindopril/amlodipine fixed-dose combination in the treatment of hypertension: a systematic review

**DOI:** 10.3389/fphar.2024.1485834

**Published:** 2024-10-17

**Authors:** Truong Van Dat, Vo Linh Tu, Le Nguyen Anh Thu, Nguyen Nhat Anh Quang, Van Binh, Nguyen Thi Quynh Nga, Duong Hoang Loc, Tran Thi Hong Nguyen, Dao Ngoc Hien Tam, Hong-Han Huynh, Tran Dinh Trung, Uyen Do, Nguyen Tuan Phat, Dang The Hung, Quang-Hien Nguyen, Nguyen Thi Hai Yen, Le Huu Nhat Minh

**Affiliations:** ^1^ Faculty of Pharmacy, University of Medicine and Pharmacy at Ho Chi Minh City, Ho Chi Minh City, Vietnam; ^2^ Faculty of Traditional Medicine, University of Medicine and Pharmacy at Ho Chi Minh City, Ho Chi Minh City, Vietnam; ^3^ Regulatory Affairs Department, Asia Shine Trading & Service Co. LTD., Ho Chi Minh City, Vietnam; ^4^ International Master Program for Translational Science, College of Medical Science and Technology, Taipei Medical University, Taipei, Taiwan; ^5^ Faculty of Public Health, Danang University of Medical Technology and Pharmacy, Danang, Vietnam; ^6^ Nelda C. Stark School of Nursing, Texas Woman’s University, Houston, TX, United States; ^7^ Cardiovascular Research Department, Methodist Hospital, Merrillville, IN, United States; ^8^ School of Biomedical Engineering & Imaging Sciences, Faculty of Life Sciences & Medicine, King’s College London, London, United Kingdom; ^9^ International Ph.D. Program in Medicine, College of Medicine, Taipei Medical University, Taipei, Taiwan; ^10^ Research Center for Artificial Intelligence in Medicine, Taipei Medical University, Taipei, Taiwan

**Keywords:** fixed-dose combination, hypertension, blood pressure, efficacy, perindopril, amlodipine, effectiveness

In the published article, there was an error in **Affiliation 2**. Instead of “Faculty of Traditional Medicine, University of Medicine and Pharmacy at Ho Chi Minh, Ho Chi Minh City, Vietnam,” it should be “Faculty of Traditional Medicine, University of Medicine and Pharmacy at Ho Chi Minh City, Ho Chi Minh City, Vietnam.”

The authors apologize for this error and state that this does not change the scientific conclusions of the article in any way. The original article has been updated.

