# Supplementary material for: Effectiveness of perindopril/amlodipine fixed-dose combination in the treatment of hypertension: a systematic review
Source: Front Pharmacol. 2024 Feb 8;14:1156655. doi: 10.3389/fphar.2023.1156655 (PMC10895423; doi:10.3389/fphar.2023.1156655)
Supplement: Supplementary file 1 [file Table1.docx]

**Supplementary Table S1. PRISMA checklist**

| **Section/topic** | **#** | **Checklist item** | **Reported on page #** | |
| --- | --- | --- | --- | --- |
| **TITLE** | | | |  |
| Title | 1 | Identify the report as a systematic review, meta-analysis, or both. |  | |
|  | | | |  |
| Structured summary | 2 | Provide a structured summary including, as applicable: background; objectives; data sources; study eligibility criteria, participants, and interventions; study appraisal and synthesis methods; results; limitations; conclusions and implications of key findings; systematic review registration number. |  | |
|  | | | |  |
| Rationale | 3 | Describe the rationale for the review in the context of what is already known. |  | |
| Objectives | 4 | Provide an explicit statement of questions being addressed with reference to participants, interventions, comparisons, outcomes, and study design (PICOS). |  | |
|  | | | |  |
| Protocol and registration | 5 | Indicate if a review protocol exists, if and where it can be accessed (e.g., Web address), and, if available, provide registration information including registration number. |  | |
| Eligibility criteria | 6 | Specify study characteristics (e.g., PICOS, length of follow-up) and report characteristics (e.g., years considered, language, publication status) used as criteria for eligibility, giving rationale. |  | |
| Information sources | 7 | Describe all information sources (e.g., databases with dates of coverage, contact with study authors to identify additional studies) in the search and date last searched. |  | |
| Search | 8 | Present full electronic search strategy for at least one database, including any limits used, such that it could be repeated. |  | |
| Study selection | 9 | State the process for selecting studies (i.e., screening, eligibility, included in systematic review, and, if applicable, included in the meta-analysis). |  | |
| Data collection process | 10 | Describe method of data extraction from reports (e.g., piloted forms, independently, in duplicate) and any processes for obtaining and confirming data from investigators. |  | |
| Data items | 11 | List and define all variables for which data were sought (e.g., PICOS, funding sources) and any assumptions and simplifications made. |  | |
| Risk of bias in individual studies | 12 | Describe methods used for assessing risk of bias of individual studies (including specification of whether this was done at the study or outcome level), and how this information is to be used in any data synthesis. |  | |
| Summary measures | 13 | State the principal summary measures (e.g., risk ratio, difference in means). |  | |
| Synthesis of results | 14 | Describe the methods of handling data and combining results of studies, if done, including measures of consistency (e.g., I^2^) for each meta-analysis. |  | |
| Risk of bias across studies | 15 | Specify any assessment of risk of bias that may affect the cumulative evidence (e.g., publication bias, selective reporting within studies). |  | |
| Additional analyses | 16 | Describe methods of additional analyses (e.g., sensitivity or subgroup analyses, meta-regression), if done, indicating which were pre-specified. |  | |
| **RESULTS** | | |  | |
| Study selection | 17 | Give numbers of studies screened, assessed for eligibility, and included in the review, with reasons for exclusions at each stage, ideally with a flow diagram. |  | |
| Study characteristics | 18 | For each study, present characteristics for which data were extracted (e.g., study size, PICOS, follow-up period) and provide the citations. |  | |
| Risk of bias within studies | 19 | Present data on risk of bias of each study and, if available, any outcome level assessment (see item 12). |  | |
| Results of individual studies | 20 | For all outcomes considered (benefits or harms), present, for each study: (a) simple summary data for each intervention group (b) effect estimates and confidence intervals, ideally with a forest plot. |  | |
| Synthesis of results | 21 | Present results of each meta-analysis done, including confidence intervals and measures of consistency. |  | |
| Risk of bias across studies | 22 | Present results of any assessment of risk of bias across studies (see Item 15). |  | |
| Additional analysis | 23 | Give results of additional analyses, if done (e.g., sensitivity or subgroup analyses, meta-regression [see Item 16]). |  | |
| **DISCUSSION** | | |  | |
| Summary of evidence | 24 | Summarize the main findings including the strength of evidence for each main outcome; consider their relevance to key groups (e.g., healthcare providers, users, and policy makers). |  | |
| Limitations | 25 | Discuss limitations at study and outcome level (e.g., risk of bias), and at review-level (e.g., incomplete retrieval of identified research, reporting bias). |  | |
| Conclusions | 26 | Provide a general interpretation of the results in the context of other evidence, and implications for future research. |  | |
| **FUNDING** | | |  | |
| Funding | 27 | Describe sources of funding for the systematic review and other support (e.g., supply of data); role of funders for the systematic review. |  | |

***Supplementary Table S2. Details of search terms in each database***

| PubMed | ("fixed dose combination" OR "fixed-dose combination" OR "fixed combination" OR "fixed-combination" OR "fixed-drug combination" OR "fixed drug combination" OR "single-pill combination" OR "single pill combination") AND (hypertension OR antihypertensive OR "blood pressure") AND perindopril AND amlodipine  Filters applied: Humans, English  n = 46 |
| --- | --- |
| Virtual Health Library | ("fixed dose combination" OR "fixed-dose combination" OR "fixed combination" OR "fixed-combination" OR "fixed-drug combination" OR "fixed drug combination" OR "single-pill combination" OR "single pill combination") AND (hypertension OR antihypertensive OR "blood pressure") AND perindopril AND amlodipine  Filters applied: Humans, English  n = 46 |
| Global Health Library | ("fixed dose combination" OR "fixed-dose combination" OR "fixed combination" OR "fixed-combination" OR "fixed-drug combination" OR "fixed drug combination" OR "single-pill combination" OR "single pill combination") AND (hypertension OR antihypertensive OR "blood pressure")  Filters applied: Humans, English  n = 15 |
| Google Scholar | with all the words: perindopril amlodipine with at least one of the words: "fixed dose combination" "fixed combination" "fixed drug combination" "single pill combination"  in the title of article  n = 81 |

***Supplementary Table S3:*** ***Baseline characteristics of included studies***

| **Author (Year)** | **Country** | **Study design** | **FDC perindopril/amlodipine dose (mg/mg)** | **Comparator** | **Follow-up period** | **n patients  (total N)** | **Age (years)** | **Male sex (%)** | **BMI (kg/m^2^)** | **Office heart rate (beats/min)** | **Washout period (days)** | **Outcome included in review** |
| --- | --- | --- | --- | --- | --- | --- | --- | --- | --- | --- | --- | --- |
| Liakos *et al*., (2017) (13) | Greece | Prospective non-interventional study | 5/5, 5/10, 10/5, and 10/10 mg ^(*)^ | none | 4 months | 1900 (1907) | 65.2 ± 10.9 | 1127 (59.1%) | 28.2 ± 3.8 | 72.2 ± 9.2 | none | Efficacy in blood pressure lowering, safety, adherence |
| Bansa *et al*., (2014) (21) | India | Multicentre prospective observational study | 4/5 mg | none | 90 days | 91 (92) | No data | No data | No data | No data | none | Efficacy in blood pressure lowering, safety |
| Manolis *et al*., (2015) (14) | Greece | Prospective observational study | 5/5, 5/10, 10/5, and 10/10 mg ^(*)^ | none | 6 months | 2231 (2300) | 64.3 ± 11.3 | 1226 (53.3%) | 28.5 ± 4.2 | No data | none | Efficacy in blood pressure lowering, safety, adherence |
| Fleig *et al.*, (2018) (17) | Germany | Prospective, non-interventional, multicenter study | 3.5/2.5 or 7.0/5.0 mg | none | 3 months | 1770 (1814) | 60.0 ± 13.4 | 973 (54.0%) | 28.9 ± 5.0 | 77.3 ± 10.1 (n = 1680) | none | Efficacy in blood pressure lowering, safety, adherence |
| Karpov *et al*., (2015) (22) | Russia | Open-label, non-comparative study | Per/Amlo, 5/5, 10/5 or 10/10 mg | none | 3 months | 86 (90) | 52.7 ± 12.2 | 59% | No data | 76.3 | none | Efficacy in blood pressure lowering, safety, adherence |
| Vlachopoulos *et al*., (2016) (15) | Greece | Prospective, non-interventional, multicenter study | 5/5, 5/10, 10/5, and 10/10 mg ^(*)^ | none | 4 months | 2254 (2269) | 65.3 ± 11.4 | 52.40% | 28.3 ± 4.2 | No data | none | Efficacy in blood pressure lowering, safety, adherence |
| Forster and Dézsi (2016) (16) | Hungary | Multicenter, prospective, observational, non-interventional, open-label data collection study | 5/5, 5/10, 10/5, and 10/10 mg ^(*)^ | none | 6 months | 3472 | 62.7 ± 9.6 | 1914 | 28.9 ± 4.8 | 7.0 ± 9.0 | none | Efficacy in blood pressure lowering, safety |
| Hatalova *et al*., (2016) (20) | Slovakia | Prospective, open-label, longitudinal, study | 5/5, 5/10, 10/5, or 10/10 mg same dose with free association at baseline | none | 3 months | 335 | 64.0 ± 10.9 | 168 (50%) | 29.1 ± 4.4 | 73.6 ± 10.1 | none | Efficacy in blood pressure lowering, safety |
| Abdelhady *et al*., (2016) (18) | Kingdom of Saudi Arabia | Observational, multicenter, open-label cohort study | 5/5, 5/10, 10/5, and 10/10 mg ^(*)^ | none | 12 weeks | 1996 (2096) | 50.9 ± 9.7 | 1417 (71%) | 30.5 ± 5.7 | No data | none | Efficacy in blood pressure lowering, safety |
| Nagy (2013) (19) | Hungary | Open, prospective, multicenter, observational study | 5/5, 5/10, 10/5, and 10/10 mg ^(*)^ | none | 3 months | 262 | 60.4 ± 11.7 | 144 (54.96%) | No data | No data | none | Efficacy in blood pressure lowering, safety |
| Ahmed *et al*., (2016) (23) | Saudi Arabia | Multi-centre, open-label, observational study | 5/5, 5/10, 10/5, and 10/10 mg ^(*)^ | none | 3 months | 582 (760) | 51 ± 10 | 506 (67%) | No data | No data | none | Efficacy in blood pressure lowering |
| Simons *et al*., (2017) (24) | Australian | Retrospective, observational study | Single-pill | Two-pill | January 2011 through December 2014 | 9340  3093 | 67.8 (67.6-68.1)  71.5 (71.0-71.9) | 4577 (49%)  1418 (46%) | No data | No data | none | Hazard ratio for discontinuation, hazard ratio for risk of death |
| Esposti *et al*., (2018) (30) | Italy | Retrospective cohort study | FDC of perindopril/amlodipine | Perindopril or amlodipine (monotherapy) or taking both drugs as free-drug combination | Two 12-month periods preceding and following the first anti-hypertensive prescription | 3597 | 65.7 ± 11.7 | 50.8% | No data | No data | none | Adherence |
| Hu *et al*., (2016) (25) | China | (Study 16) Phase III randomized double-blind trials | 5/5 mg for 8 weeks, at week 8, up-titrated to 5/10 mg for the patients who had not reached the target BP | Amlodipine 5 mg for 8 weeks, at week 8, added perindopril 5 mg for the patients who had not reached the target BP | 12 weeks | 455 (492) | 51.1 ± 10.1  52.5 ± 9.1 | 139 (56.3%)  135 (55.1%) | 26.1 ± 2.4  25.8 ± 2.5 | No data | Amlodipine 5 mg once daily for 4 weeks | Efficacy in blood pressure lowering, safety |
|  |  | (Study 17) Phase III randomized double-blind trials | 5/5 mg for 8 weeks, at week 8, up-titrated to 10/5 mg for the patients who had not reached the target BP | Perindopril 4 mg for 8 weeks, at week 8, switched to Perindopril 5 mg + amlodipine 5 mg for the patients who had not reached the target BP | 12 weeks | 332 (353) | 53.7 ± 9.0  52.7 ± 9.0 | 103 (57.9%)  91 (52.0%) | 26.2 ± 2.3  25.2 ± 2.6 | No data | Perindopril 4 mg once daily for 4 weeks |  |
| Laurent *et al*., (2015) (29) | 6 European countries | International, randomized, double-blind, placebo-controlled study | 3.5/2.5 mg | Monotherapy (Perindopril 3.5 mg, Amlodipine 2.5 mg, Perindopril 5 mg, Amlodipine 5 mg), placebo | 8 weeks | 1497 (1581) | 51.7 ± 11.4 | 739 (46.7%) | 26.8 ± 2.6 | No data | none | Efficacy in blood pressure lowering, safety |
| Sobngwi *et al*., (2019) (26) | Cameroon | Double‐blinded randomized controlled trial | 5/5 mg | Perindopril/indapamide FDC 5/1.25 mg | 6 weeks | 15 patients each group | 57 [53‐60]  60 [52‐64] | 7 males each group | 29 [26-31]  28 [27‐33] | 80 [75 -85]  79 [73‐84] | none | Efficacy in blood pressure lowering, safety |
| Mancia *et al*., (2015) (28) | 18 countries | Double-blind, parallel-group, randomized controlled trial | 3.5/2.5 mg, uptitration 7/5 mg, 14/10 mg | Valsartan 80 mg, uptitration valsartan 160 mg, valsartan/amlodipine 160/5 mg | 3 months  (Data collected) | 834 (888)  828 (886) | 55.7 ± 10.3  55.2 ± 10.9 | 479 (54%)  469 (53%) | 27.1 ± 2.4  27.1 ± 2.5 | No data | none | Efficacy in blood pressure lowering, safety |

^(*)^ according to the approved summary of product characteristics for the management of arterial hypertension and CAD

***Supplementary Table S4:*** ***Effectiveness and cost-effectiveness of perindopril/amlodipine fixed-dose combination in the treatment of hypertension***

| **Author (Year)** | **Follow-up period** | **Intervention or Comparator** | **Results** | | **Safety** | **Adherence** | **Conclusion** |
| --- | --- | --- | --- | --- | --- | --- | --- |
|  |  |  | **Baseline**  **(mean ± SD)** | **Last visit**  **(mean ± SD)** |  |  |  |
| Liakos *et al*., (2017) (13) | 4 months | Per/Amlo FDC 5/5, 5/10, 10/5, and 10/10 mg ^(*)^ | Average SBP/DBP was 156.5 ± 15.0/89.9 ± 9.6 mmHg  Uncontrolled hypertension was 90.3% | Average SBP/DBP was 130.8 ± 8.4/78.2 ± 6.4 mmHg (p < 0.001 vs. baseline)  Uncontrolled hypertension was 18.5% | 13 (0.7%) patients reported non-serious ADRs: lower limb edema (n=11), cough (n = 1), extra-systolic arrhythmia (n = 1) | Average adherence was 0.8 ± 0.4 | Perindopril/amlodipine FDC was characterized by high adherence and effectiveness, regardless of previous treatment |
| Bansa *et al*., (2014) (21) | 90 days | Per/Amlo FDC 4/5 mg | Hypertension stage 2, 3 | Stage 2 hypertension SBP/DBP decreased 35.2 (31.7-38.7)/20.6 (19.2-22.1) mmHg  Stage 3 hypertension SBP/DBP decreased 46.1 (40.1-52.3)/23.8 (19.9-27.7) mmHg | Dry cough (n = 4, 1 withdrew)  Ankle edema (n = 3) | No data | Perindopril’s FDC with amlodipine reduces blood pressure effectively, resulting in high rates of blood pressure control over the short term, with a low frequency of side effects including cough and pedal edema |
| Manolis *et al*., (2015) (14) | 6 months | Per/Amlo FDC 5/5, 5/10, 10/5, and 10/10 mg ^(*)^ | Average SBP/DBP was 157.0±15.4/91.5±10.1 mmHg | Average SBP/DBP was 129.0±7.9/78.8±6.7 mmHg (p < 0.001 vs. baseline)  BP control (<140/90 mm Hg) was achieved in 84.8% (1893/2231) | Adverse events/reactions leading to treatment discontinuation (n = 27): 21 minor adverse events (e.g. cough, ankle, or lower limb edema), 6 serious adverse events (1 cases of face edema) | Most patients (n = 2233; 97.1%) took perindopril/amlodipine “every day” or “quite often” | Fixed-dose combination perindopril/amlodipine safely reduced and controlled elevated BP in patients with essential hypertension in a real-life clinical setting, with good patient compliance |
| Fleig *et al.*, (2018) (17) | 3 months | Per/Amlo FDC 3.5/2.5 or 7.0/5.0 mg | Average SBP/DBP was 163.7 ± 14.8/ 95.4 ± 9.4 mmHg (n = 1770)  Grade 3 hypertension: 21.3% (n = 377); grade 2 hypertension: 51.1% (n = 905), grade 1 hypertension 26.1% (n = 462) | Average SBP/DBP was 133.6 ± 11.6/ 80.3 ± 7.7 mmHg (n = 1770) (p < 0.0001 vs. baseline)  Normal or high normal blood pressure: 69.1% (n = 1223), grade 1 hypertension: 25.7% (n = 455), grade 2 hypertension: 3.8% (n = 68), grade 3 hypertension: 1.4% (n = 24) | 140 ADRs were reported for 88 patients (4.9%) (seriousness was confirmed for ADR in 8 patients (0.4%)), ADR was reported to be recovered for 135 events in 83 patients, not recovered for 4 patients (0.2%), and fatal for one event in 1 patient with a hypertensive crisis.  Tolerability of perindopril/amlodipine was rated by the treating physician as ‘‘very good’’ (n = 1308/1724; 75.9%) or ‘‘good’’ (n = 392/1724; 22.7%) | 791/1676 (47.2%) patients showed perfect adherence at the final visit | Fixed-dose combination of perindopril/amlodipine showed significant blood pressure reduction and improvement in medication adherence in a primary care setting |
| Karpov *et al*., (2015) (22) | 3 months | Per/Amlo FDC 5/5, 10/5 or 10/10 mg | Average 24h SBP/DBP was 152.8 ± 10.5/90.0 ± 7.5 mmHg  Average SBP/DBP was 161.4/94.9 mmHg | Average 24h SBP/DBP was 125.6 ± 25.2/81.0 ± 11.1 mmHg (p < 0.0001 vs. baseline)  Average SBP/DBP decreased significantly by 33.7/17.1 mmHg from baseline (p < 0.001 vs. baseline) | No withdrawal was due to AEs. 12 AEs occurred, 8 cases of lower limb edema | Increased from 2.65 ± 1.19 points at baseline to 3.57 ± 0.75 points by 3 months (Morisky-Green scale) | FDC perindopril/amlodipine resulted in a rapid and pronounced antihypertensive effect, with target BP levels achieved after 3 months in most patients |
| Vlachopoulos *et al*., (2016) (15) | 4 months | Per/Amlo FDC 5/5, 5/10, 10/5, and 10/10 mg ^(*)^ | Average SBP/DBP was 158.4 ± 13.6/89.9 ± 8.7 mmHg | Average SBP/DBP was 130.3 ± 7.9/77.7 ± 6.3 mmHg (p < 0.001 vs. baseline)  BP control (<140/90 mm Hg) was achieved in 88.5% (of 2254) | 25 patients presented adverse events: 15 patients minor adverse (lower limb edema (n = 8), dry cough (n = 4), hypotension (n = 3)); 9 patients presented adverse events leading to treatment discontinuation (cough (n = 3), lower limb edema (n = 3), hypotension (n = 2), constipation (n = 1) | 98.3% were taking their treatment “every day” or “quite often” | Perindopril/amlodipine FDC decreased BP levels promptly and significantly with a low grade of visit-to-visit variability. Simplifying the drug regime by using this FDC improved adherence and tolerability, an important requirement for the improvement of prognosis in these patients |
| Forster and Dézsi (2016) (16) | 6 months | Per/Amlo FDC 5/5, 5/10, 10/5, and 10/10 mg ^(*)^ | Average SBP/DBP was 157.5 ± 12.9/92.9 ± 8.6 mmHg | Average SBP/DBP was 130.3 ± 8.3/79.8 ± 6.1 mmHg (p < 0.0001 vs. baseline)  BP target value was reached by 80.6% of patients  Mean heart rate decreased by 7.0 ± 9.0 bpm (p < 0.0001)  The exercise tolerance of the patients increased significantly: their mean maximal performance increased from 88.9 ± 37.9 to 110.5 ± 38.4 W (+24.4%; p < 0.001) and from 7.86 ± 2.95 to 8.78 ± 2.92 MET (+11.7%; p < 0.001) (n =197) | The most frequent ones of the recorded 39 events in 37 patients: crural edema and ankle edema 24 (0.6%), coughing 7 (0.2%), and hypotension 4 (0.1%) | No data | The fixed-dose combination of perindopril and amlodipine could have favorable effects on the cardiovascular system, not only by its BP-lowering effect and its effect on vascular resistance but also through its direct cardiovascular protective effects. |
| Hatalova *et al*., (2016) (20) | 3 months | 5/5, 5/10, 10/5, or 10/10 mg same dose with free association at baseline | Average SBP/DBP was 153.4 ± 15.9/91.5 ± 9.5 mmHg | Mean change from baseline was -23.3 ± 16.4/-11.3 ± 9.8 mmHg at 3 months (p < 0.0001)  The percentage of patients at or below blood pressure target increased from 16.0 % at baseline to 75.9 % at 3 months  Heart rate decreases -3.5 ± 7.9 beats/min (p < 0.0001) | No serious adverse events were reported. The most frequently observed adverse events reported were ankle edema (14.9 %), dyspnea (0.6 %), headache (0.6 %), back pain (0.6 %), and vertigo (0.3 %). The relative risk reduction for ankle edema was -37.5 % at 1 month (vs. baseline; p < 0.001) and -57.2 % at 3 months (vs. baseline; p < 0.001) | No data | The FDC formulation of perindopril/amlodipine was particularly well adapted to daily practice as it led to significant improvements in efficacy and tolerability compared to free associations of the same doses |
| Abdelhady *et al*., (2016) (18) | 12 weeks | Per/Amlo FDC 5/5, 5/10, 10/5, and 10/10 mg ^(*)^ | Average SBP/DBP was 161.7 ± 13.9/99.4 ± 8.4) mmHg | Average SBP/DBP was 127.6 ± 8.6/80.1 ± 5.4 mmHg (p < 0.0001 vs. baseline)  93.3 % of patients having controlled BP at week 12 | Ankle edema (16%), mild cough (5%), and headache with dizziness (4%) of 2096 | No data | Fixed combination of perindopril/amlodipine was effective in controlling BP in patients with essential hypertension with an overall 93.3 % control of both SBP and DBP |
| Nagy (2013) (19) | 3 months | 5/5, 5/10, 10/5, and 10/10 mg ^(*)^ | Average SBP/DBP was 159.8 ± 16.0/94.3 ± 10.3 mmHg  Average 24h SBP/DBP was 146.1 ± 15.3/84.3 ± 11.3 mmHg | Mean office BP decreased to 131.1 ± 10.2/80.0 ± 7.1 (p < 0.001)  Mean 24h ambulatory BP decreased to 127.6/75.9 mmHg (p < 0.001) | No serious adverse events were reported. 7 adverse events occurred: swelling of the lower extremities (n = 4), cough (n = 1), angioedema (n = 1), bradycardia (n =1, but not related to treatment) | No data | Perindopril/amlodipine fixed-dose combination could be considered an effective and safe option to normalize BP |
| Ahmed *et al*., (2016) (23) | 3 months | 5/5, 5/10, 10/5, and 10/10 mg ^(*)^ | Average SBP/DBP was 160/98 mmHg | SBP decreased 31 mmHg (p <0.001) and DBP decreased 18 mmHg (p < 0.001)  BP control rate was achieved in 87% (n=507) | No data | No data | The perindopril/amlodipine combination was an effective and well tolerated anti-hypertensive option in patients on previous ARB treatment |
| Simons *et al*., (2017) (24) | January 2011 through December 2014 | Single-pill | No data | 8% of single-pill users and 18% of two-pill users had died  the hazard ratio for risk of death in the two-pill versus single-pill group was 2.81 (95%ci 2.42-3.26) | No data | After 12 months, 34% of single-pill and 57% of two-pill users discontinued | Use of a single-pill, fixed-dose combination in hypertension was associated with superior persistence and reduced mortality compared with use of two pills of the individual drugs |
| Esposti *et al*., (2018) (30) | Two 12-month periods preceding and following the first anti-hypertensive prescription | FDC of perindopril/amlodipine | No data | No data | No data | The percentage of individual adherence observed significantly (from 59.3% to 72.2%, +22%, p < 0.001) | The single-pill, FDC of perindopril/amlodipine was associated with significant higher rates of adherence to treatment than other anti-hypertensive strategies |
| Hu *et al*., (2016) (25) | 12 weeks (study 16) | 5/5 mg for 8 weeks, at week 8, up-titrated to 5/10 mg for the patients who had not reached the target BP | Average SBP/DBP was 149.6 ± 6.9/96.9 ± 4.6 mmHg (n = 247) | Mean SBP dropped by 11.1 ± 11.9 mmHg in the Per/Amlo group and by 8.5 ± 11.1 mmHg in the Amlo group (between-group difference: –2.7 mmHg, 95% CI: –4.7 to –0.7; p = 0.0095) at W8, Mean SBP decreased from W8 of –8.2 ± 11.8 mmHg in the up-titrated subgroup Per/Amlo group and –6.1 ± 11.4 mmHg in the up-titrated subgroup Amlo group at W12 | 49 (19.8%) after 8w and 33 (24.6%) w8-w12 (3 serious EAEs in 3 patients, 1.2% (ischaemic stroke (n = 1), synovitis (n = 1)) at W8 and of 3 serious EAEs in the up-titrated patients during the period W8–W12). Non-serious EAEs leading to treatment discontinuation: cough (n = 3) | No data | Single-pill combinations of perindopril and amlodipine provided hypertensive patients with a convenient and effective method of reducing blood pressure |
|  |  | Amlodipine 5 mg for 8 weeks, at week 8, added perindopril 5 mg for the patients who had not reached the target BP | Average SBP/DBP was 150.5 ± 7.4/96.4 ± 4.4 mmHg (n = 245) |  | 31 (12.7%) after 8w and 39 (25.7%) w8-w12 (1 serious EAE of lumbar intervertebral disc protrusion) |  |  |
|  | 12 weeks (study 17) | 5/5 mg for 8 weeks, at week 8, up-titrated to 10/5 mg for the patients who had not reached the target BP | Average SBP/DBP was 150.7 ± 6.8/97.6 ± 5.0 mmHg (n = 178) | Mean SBP dropped by 15.8 ± 12.1 mmHg in the Per/Amlo group and by 7.8 ± 13.7 mmHg in the Per group (between-group difference: –7.6 mmHg, 95% CI: –10.3 to –5.0; p < 0.0001) at W8, mean SBP decreased from W8 of –4.8 ± 11.3 mmHg in the up-titrated subgroup Per/Amlo group and –12.2 ± 11.5 mmHg in the up-titrated subgroup Per group at W12 | 34 (19.1%) after 8w and 11 (14.5%) w8-w12 (1 serious EAE of diaphragmatic eventration). Non-serious EAEs leading to treatment discontinuation: cough (n = 2) |  |  |
|  |  | Perindopril 4 mg for 8 weeks, at week 8, switched to Perindopril 5 mg + amlodipine 5 mg for the patients who had not reached the target BP | Average SBP/DBP was 150.0 ± 6.8/97.7 ± 4.7 mmHg (n = 175) |  | 23 (13.1%) after 8w and 13 (11.7%) w8-w12. Non-serious EAEs leading to treatment discontinuation: cough (n = 2), itch (n = 1) |  |  |
| Laurent *et al*., (2015) (29) | 8 weeks | Per/Amlo FDC 3.5/2.5 mg | Average SBP/DBP was 161.4 ± 7.5/100.5 ± 4.0 mmHg | The decrease in SBP was greater in the Per/Amlo FDC 3.5/2.5 mg group (22.0 mmHg) than with placebo (14.2 mmHg), perindopril 3.5 mg (16.3 mmHg), amlodipine 2.5 mg (16.0 mmHg) with a significant between-group difference of -7.22 mmHg (p < 0.001), -5.01 mmHg (p < 0.001) and -5.20 mmHg (p < 0.001), respectively. The combination was significantly non-inferior to both of the component drugs administered at their lowest clinically approved doses (p < 0.001 versus perindopril 5 mg, and p = 0.003 versus amlodipine 5 mg).  The decrease in DBP was greater in the Per/Amlo FDC 3.5/2.5 mg group (13.6 mmHg) than with placebo (9.3 mmHg), perindopril 3.5 mg (9.3 mmHg), amlodipine 2.5 mg (10.3 mmHg) with a significant between-group difference of -4.12 mmHg (p < 0.001), -3.64 mmHg (p < 0.001) and -2.97 mmHg (p < 0.001), respectively. The combination was therefore significantly non-inferior to both of the component drugs administered at their lowest clinically approved doses (p < 0.001 in both cases) | The incidence of all EAEs in the combination group was 18.9%, slightly higher than with placebo (15.9%) and similar to the perindopril 3.5 mg (18.7%) and the amlodipine 2.5 mg (18.6%) groups.  Serious EAEs were infrequent: there were no cases in the combination group and one case with placebo, compared with four and three cases in the perindopril 5 mg and amlodipine 5 mg groups, respectively | No data | The combination perindopril 3.5 mg/amlodipine 2.5 mg produced significant and clinically relevant decreases in blood pressure relative to placebo in patients with uncomplicated mild-to- moderate hypertension. The combination was superior to either component given singly, and was also non-inferior to the component drugs given singly at their lowest approved doses, using guideline-recommended criteria for clinical relevance and non-inferiority |
|  |  | Monotherapy (Perindopril 3.5 mg, Amlodipine 2.5 mg, Perindopril 5 mg, Amlodipine 5 mg), placebo |  |  |  |  |  |
| Sobngwi *et al*., (2019) (26) | 6 weeks | Per/Amlo FDC 5/5 mg (n = 15) | Average 24h SBP/DBP was 144[138‐152]/ 85[75‐89] mmHg  Average SBP/DBP was 144 [140‐150]/91[85‐93] mmHg | Average 24h SBP/DBP was 128[125‐138]/78[73‐83] mmHg (p = 0.003 (SBP), p = 0.013 (DBP) vs. baseline)  Average SBP/DBP was 128[119‐133]/73[72‐82] mmHg (p = 0.03 (SBP), p = 0.001 (DBP) vs. baseline; p = 0.7 (SBP), p = 0.6 (DBP) vs. Comparator) | No significant adverse effect was reported during intervention | No data | Fixed combination therapies with perindopril‐amlodipine and perindopril‐indapamide were effective for blood pressure control after 6 weeks when used to initiate antihypertensive treatment in type 2 diabetic patients |
|  |  | Perindopril/indapamide FDC 5/1.25 mg (n = 15) | Average 24h SBP/DBP was 145[137‐155]/89[82‐93] mmHg  Average SBP/DBP was 147[146‐151]/89[84‐96] mmHg | Average 24h SBP/DBP was 126[116‐137]/79[68‐86] mmHg (p = 0.003 (SBP), p = 0.006 (DBP) vs. baseline)  Average SBP/DBP was 124 [108‐137]/ 78 [70‐89] mmHg (p = 0.008 vs. baseline) |  |  |  |
| Mancia *et al*., (2015) (28) | 3 months (Data collected) | 3.5/2.5 mg, uptitration 7/5 mg, 14/10 mg | Average SBP/DBP was 163.6 ± 7.9/100.2 ± 3.7 mmHg | Average SBP/DBP was 137.8 ± 12.4/ 83.3 ± 8.7 mmHg  Greatest reductions from baseline with perindopril/amlodipine (primary endpoint -2.0/-1.5 mmHg; both p < 0.001 vs. Comparator) | The safety of the two strategies was equivalent | No data | The three-step single-pill antihypertensive strategy of perindopril/amlodipine (3.5/2.5, 7/5, and 14/10 mg daily) produced greater reductions in blood pressure, and better and quicker rates of control of hypertension than a single-pill combination of valsartan/amlodipine |
|  |  | Valsartan 80 mg, uptitration valsartan 160 mg, valsartan/amlodipine 160/5 mg | Average SBP/DBP was 163.4 ± 8.0 /100.2 ± 3.8 mmHg | Average SBP/DBP was 139.7 ± 13.3/ 84.8 ± 9.0 mmHg |  |  |  |

^(*)^ according to the approved summary of product characteristics for the management of arterial hypertension and CAD

***Supplementary Table S5:*** ***Quality assessment for cohort and cross-sectional studies***

| **References** | | **Question** | | | | | | | | | | | | | | **Overall** |
| --- | --- | --- | --- | --- | --- | --- | --- | --- | --- | --- | --- | --- | --- | --- | --- | --- |
| **No** | **Article** | **1** | **2** | **3** | **4** | **5** | **6** | **7** | **8** | **9** | **10** | **11** | **12** | **13** | **14** |  |
| 1 | Liakos *et al*., (2017) (13) | 1 | 1 | 1 | 1 | 0 | 1 | 0 | 1 | 1 | 1 | 1 | 0 | 1 | 1 | Good |
| 2 | Bansa *et al*., (2014) (21) | 1 | 1 | 1 | 1 | 0 | 1 | 1 | 1 | NA | 1 | 1 | 0 | 1 | 1 | Good |
| 3 | Manolis *et al*., (2015) (14) | 1 | 1 | 1 | 1 | 1 | 1 | 1 | 1 | 1 | 1 | 1 | 0 | 1 | 1 | Good |
| 4 | Fleig *et al.*, (2018) (17) | 1 | 1 | 1 | 0 | 0 | 1 | 1 | 1 | 1 | 1 | 1 | 0 | 1 | 1 | Good |
| 5 | Karpov *et al*., (2015) (22) | 1 | 1 | 1 | 1 | 0 | 1 | 1 | 1 | 1 | 1 | 1 | 0 | 1 | 1 | Good |
| 6 | Vlachopoulos *et al*., (2016) (15) | 1 | 1 | 1 | 1 | 0 | 1 | 1 | 1 | 1 | 1 | 1 | 0 | 1 | 1 | Good |
| 7 | Simons *et al*., (2017) (24) | 1 | NA | 1 | 0 | 1 | 1 | 1 | 1 | 1 | 1 | 1 | 0 | 1 | NA | Good |
| 8 | Esposti *et al*., (2018) (30) | 1 | 1 | 1 | 1 | 0 | 1 | 1 | 1 | 1 | 1 | 1 | 0 | NA | 1 | Good |
| 9 | Forster and Dézsi (2016) (16) | 1 | 1 | 1 | 1 | 0 | 1 | NA | 1 | NA | 1 | 1 | 0 | 1 | 1 | Good |
| 10 | Hatalova *et al*., (2016) (20) | 1 | 1 | 0 | 1 | 0 | 1 | 1 | 1 | 1 | 1 | 1 | 0 | 1 | 1 | Good |
| 11 | Abdelhady *et al*., (2016) (18) | 1 | 1 | 1 | 1 | 0 | 1 | 1 | 1 | 1 | 1 | 1 | 0 | 1 | 1 | Good |
| 12 | Nagy (2013) (19) | 1 | 1 | 1 | 1 | 0 | 1 | 1 | 1 | 1 | 1 | 1 | 0 | 1 | 1 | Good |
| 13 | Ahmed *et al*., (2016) (23) | 1 | 1 | 1 | 1 | 0 | 0 | 1 | 0 | 1 | 1 | 1 | 0 | 0 | 1 | Fair |

Question 1. Was the research question or objective in this paper clearly stated?

Question 2. Was the study population clearly specified and defined?

Question 3. Was the participation rate of eligible persons at least 50%?

Question 4. Were all the subjects selected or recruited from the same or similar populations (including the same time periods)? Were inclusion and exclusion criteria for being in the study prespecified and applied uniformly to all participants?

Question 5. Was a sample size justification, power description, or variance and effect estimates provided?

Question 6. For the analyses in this paper, were the exposure(s) of interest measured prior to the outcome(s) being measured?

Question 7. Was the timeframe sufficient so that one could reasonably expect to see an association between exposure and outcome if it existed?

Question 8. For exposures that can vary in amount or level, did the study examine different levels of the exposure as related to the outcome (e.g., categories of exposure, or exposure measured as continuous variable)?

Question 9. Were the exposure measures (independent variables) clearly defined, valid, reliable, and implemented consistently across all study participants?

Question 10. Was the exposure(s) assessed more than once over time?

Question 11. Were the outcome measures (dependent variables) clearly defined, valid, reliable, and implemented consistently across all study participants?

Question 12. Were the outcome assessors blinded to the exposure status of participants?

Question 13. Was loss to follow-up after baseline 20% or less?

Question 14. Were key potential confounding variables measured and adjusted statistically for their impact on the relationship between exposure(s) and outcome(s)?

***Supplementary Table S6:*** ***Quality assessment for RCT using ROB2 from Cochrane***

| **Reference ID** | **Domain 1: Risk of bias arising from the randomization process** | **Domain 2: Risk of bias due to deviations from the intended interventions (effect of assignment to intervention)** | **Domain 2: Risk of bias due to deviations from the intended interventions (effect of adhering to intervention)** | **Domain 3: Missing outcome data** | **Domain 4: Risk of bias in measurement of the outcome** | **Domain 5: Risk of bias in selection of the reported result** | **Domain 6: Overall bias** |
| --- | --- | --- | --- | --- | --- | --- | --- |
| Hu *et al*., (2016) (25) | Low | Low | Some concerns | Low | Low | Low | Some concerns |
| Laurent *et al*., (2015) (29) | Low | Low | Some concerns | Low | Low | Low | Some concerns |
| Sobngwi *et al*., (2019) (26) | Low | Low | Some concerns | Low | Low | Low | Some concerns |
| Mancia *et al*., (2015) (28) | Low | Low | Some concerns | Low | Low | Low | Some concerns |

***Supplementary Table S6. Details of search terms in each database***

| PubMed | ("fixed dose combination" OR "fixed-dose combination" OR "fixed combination" OR "fixed-combination" OR "fixed-drug combination" OR "fixed drug combination" OR "single-pill combination" OR "single pill combination") AND (hypertension OR antihypertensive OR "blood pressure") AND perindopril AND amlodipine  Filters applied: Humans, English  n = 46 |
| --- | --- |
| Virtual Health Library | ("fixed dose combination" OR "fixed-dose combination" OR "fixed combination" OR "fixed-combination" OR "fixed-drug combination" OR "fixed drug combination" OR "single-pill combination" OR "single pill combination") AND (hypertension OR antihypertensive OR "blood pressure") AND perindopril AND amlodipine  Filters applied: Humans, English  n = 46 |
| Global Health Library | ("fixed dose combination" OR "fixed-dose combination" OR "fixed combination" OR "fixed-combination" OR "fixed-drug combination" OR "fixed drug combination" OR "single-pill combination" OR "single pill combination") AND (hypertension OR antihypertensive OR "blood pressure")  Filters applied: Humans, English  n = 15 |
| Google Scholar | with all the words: perindopril amlodipine with at least one of the words: "fixed dose combination" "fixed combination" "fixed drug combination" "single pill combination"  in the title of article  n = 81 |
